# Internet addiction in adolescents with suicidal ideation: the role of self-esteem and school connectedness

**DOI:** 10.3389/fpsyt.2026.1775949

**Published:** 2026-04-01

**Authors:** Haotian Chen, Haiqin Chen, Jianfeng Xu, Yue Mao, Fei Yin, Jingfen Jin

**Affiliations:** 1 Nursing Department, The Second Affiliated Hospital of Zhejiang University School of Medicine (SAHZU), Hangzhou, Zhejiang, China; 2 School of Nursing, Li Ka Shing Faculty of Medicine, The University of Hong Kong, Hong Kong, China; 3 Nursing Department, Huzhou Third Municipal Hospital, Affiliated Hospital of Huzhou University, Huzhou, Zhejiang, China; 4 Key Laboratory of Basic Research and Health Management on Chronic Diseases, Harbin Medical University, Daqing, Heilongjiang, China

**Keywords:** adolescents, internet addiction, mediator, moderator, school connectedness, self-esteem, suicidal ideation

## Abstract

**Background:**

Internet addiction (IA) has become a growing concern, particularly among adolescents, due to its adverse effects on mental health, physical well-being, and future development. Adolescents with suicidal ideation (SI) are particularly vulnerable to IA, which may be associated with a higher risk of engaging in suicidal behaviors. However, the relationship and underlying mechanisms between SI and IA remain unclear. This study, grounded in the cognitive-behavioral model of pathological internet use, investigates the relationship and explores the roles of self-esteem (mediator) and school connectedness (moderator) in this association.

**Methods:**

In this cross-sectional study, 462 Chinese adolescents with SI (79.0% female) were recruited from psychiatric outpatient clinics between June 2024 and September 2025. Validated instruments measured SI, self-esteem, school connectedness, and IA. Structural equation modeling with bootstrapping procedures was used to test the mediation effect of self-esteem on the relationship between SI and IA. The moderating role of school connectedness was examined using PROCESS Model 8.

**Results:**

SI was positively associated with IA (β = 0.224, p < 0.001). SI was negatively associated with self-esteem (β = -0.464, p < 0.001), and self-esteem was further negatively associated with IA (β = -0.448, p < 0.001). Self-esteem partially mediated the relationship between SI and IA, with an indirect effect of 0.208 (95% CI: 0.154-0.271). School connectedness significantly moderated the direct association between SI and IA (β = -0.005, p = 0.001), but did not moderate the association between SI and the mediator, self-esteem (β = 0.004, p = 0.202).

**Conclusion:**

This study identifies a significant positive association between SI and IA among adolescents with SI, with self-esteem partially mediating this link. Furthermore, school connectedness showed a very weak buffering effect on the direct association between SI and IA, and it does not moderate the association between SI and self-esteem. These findings enhance our understanding of the mechanisms underlying IA in this vulnerable population and suggest potential targets for interventions.

## 1 Introduction

Internet addiction (IA) is defined as the excessive or uncontrollable use of the internet, leading to symptoms such as withdrawal and tolerance (1). It is also referred to as “problematic internet use”, “compulsive internet use”, and “pathological internet use”. Adolescence, a period of intense psychological development and change, is when individuals are not only most adept at adopting and adapting to the internet but also most vulnerable to developing IA compared with other age groups (2, 3). Studies indicate that approximately 21.6% of adolescents in South Korea (4), 13.0% in China (5), and 5.1% in European countries (6) suffer from IA. IA significantly affects various aspects of adolescent development, including mental and physical health, such as increasing the risk of depression (7), anxiety (8), and attention deficit hyperactivity disorder (3), as well as academic performance (9). The DSM-5 has classified IA as a behavioral addiction, categorizing it under gaming disorder (10), highlighting the critical need for addressing IA as a significant issue affecting adolescents.

Recent studies have shown that adolescents with suicidal ideation (SI) are more vulnerable to problematic smartphone use than their peers, with a 2- to 2.6-fold increased risk (11, 12). A study of 439 Chinese college students found that those with SI were more likely to spend over five hours a day on their phones and have more severe mobile phone addiction (11). This phenomenon suggests that adolescents with SI may be particularly vulnerable to IA. This compounding association is particularly concerning because IA is negatively associated with the core mechanisms that buffer the transition from SI to SAs, such as executive function (13), impulse control (14), and emotional regulation (15). These IA-related impacts severely affect adolescents with SI, pushing them closer to the precipice of SAs. However, whether and how SI is associated with IA among this specific population has not been thoroughly examined. Exploring these associative pathways not only helps to reveal the phenomenon of IA among adolescents with SI but also contributes to a better understanding of the mechanisms involved, which could inform future interventions aimed at addressing both conditions.

To understand this phenomenon, IA should be situated within the context of social and cognitive-behavioral processes (16, 17). The Cognitive-Behavioral Model of Pathological Internet Use (CBM-PIU), introduced by Davis (18), is the most widely cited framework for conceptualizing IA. According to the CBM-PIU, IA is jointly promoted by three core elements: underlying psychopathology acting as a distal vulnerability, proximal maladaptive cognitions about the self, and a concurrent lack of offline social support (18). Previous studies have predominantly focused on IA as a significant risk factor for SI (19) and suicidal attempts (20). However, recent evidence indicates that SI is significantly associated with a higher risk of IA (11, 12). Rather than merely being a passive outcome, SI may act as a critical vulnerability factor. Adolescents struggling with SI may turn to excessive internet use as a maladaptive coping strategy to escape overwhelming negative emotions and psychological pain (21). Therefore, aligning with this evidence, we propose our first hypothesis:

Hypothesis 1: SI is positively associated with IA among adolescents with SI.

As CBM-PIU suggested, proximal maladaptive self-cognitions serve as the core internal mechanisms directly maintaining IA (18). Self-esteem, defined as the degree to which an individual perceives their own self-worth (22), represents this fundamental internal cognition. Low self-esteem is associated with various maladaptive behaviors in adolescents, including aggressive behavior (23, 24), risky sexual behavior (25), and eating disorder behavior (26). Systematic review results indicate that fulfilling self-esteem needs is a central cognitive process underlying IA, as the online environment offers a sense of mastery, accomplishment, and autonomy that compensates for deficiencies in self-worth (17). Consistent with this review, self-esteem has been shown to be negatively associated with IA (27, 28) and to mediate the association between various psychosocial factors, such as family atmosphere, and IA (29).

Self-esteem is also strongly linked to SI. Numerous studies have demonstrated that low self-esteem is a significant predictor of more severe SI (30, 31). However, recent research suggests that this relationship may be bidirectional, meaning that self-esteem is not only associated with the development of SI but that SI is also linked to lower self-esteem. For example, a longitudinal study among Chinese adolescents found that those with high SI were more likely to have lower self-esteem at subsequent time points (32). According to the Sociometer Theory of Self-esteem, individuals’ self-esteem fluctuates based on perceived acceptance or rejection by others in their social environment (33). Adolescents with SI are particularly sensitive to negative social rejection cues, which further erode their self-esteem (34). Although this suggests a potential mechanism, the indirect association between SI and IA via self-esteem has not yet been examined. Therefore, we propose the following hypotheses:

Hypothesis 2a: SI is negatively related to lower self-esteem.

Hypothesis 2b: Self-esteem is negatively related to IA.

Hypothesis 2c: Self-esteem mediates the association between SI and IA among adolescents with SI.

In addition to internal cognitions, the CBM-PIU posits that inadequate offline social support exacerbates the drive for online emotional compensation (18). As primary sources of this offline support, the school and family are the most proximal contexts that influence adolescent behavioral development (35). Compared to the family environment, the school environment offers an ideal setting for large-scale interventions, serving as a compensatory source of support, especially for adolescents from disadvantaged family backgrounds (36).

School connectedness, defined as an adolescent’s subjective sense of belonging within the school environment and the strength of meaningful relationships with teachers and peers, serves as a core indicator of this school-based support (37). Previous studies have demonstrated that school connectedness plays a protective role in adolescent mental health (38). Specifically, higher levels of school connectedness are negatively correlated with SI, with adolescents who report stronger connections to their school being less likely to experience SI or attempt suicide (39, 40). Moreover, Chang et al. (41) found that children with negative school bonds were more likely to develop IA. These findings suggest that school connectedness may serve as a protective factor, buffering adolescents with SI from developing IA.

Based on previous studies, school connectedness is generally considered an essential moderator against adverse factors (2, 42). For example, it moderates the association between interparental conflict and adolescent IA, with this relationship being weaker for adolescents with higher levels of school connectedness (43). Additionally, school connectedness has been found to protect self-esteem from negative influences, such as parental rejection (44). However, it remains unclear whether school connectedness functions as a moderator in the association between SI and IA, and whether this moderating role is mediated through the hypothesized mediator, self-esteem. Therefore, we propose the following hypotheses:

Hypothesis 3a: School connectedness moderates the effect of SI on IA, such that higher levels of school connectedness attenuate the relationship between SI and IA.

Hypothesis 3b: School connectedness moderates the effect of SI on self-esteem, such that higher levels of school connectedness attenuate the relationship between SI and self-esteem.

**Figure 1:**
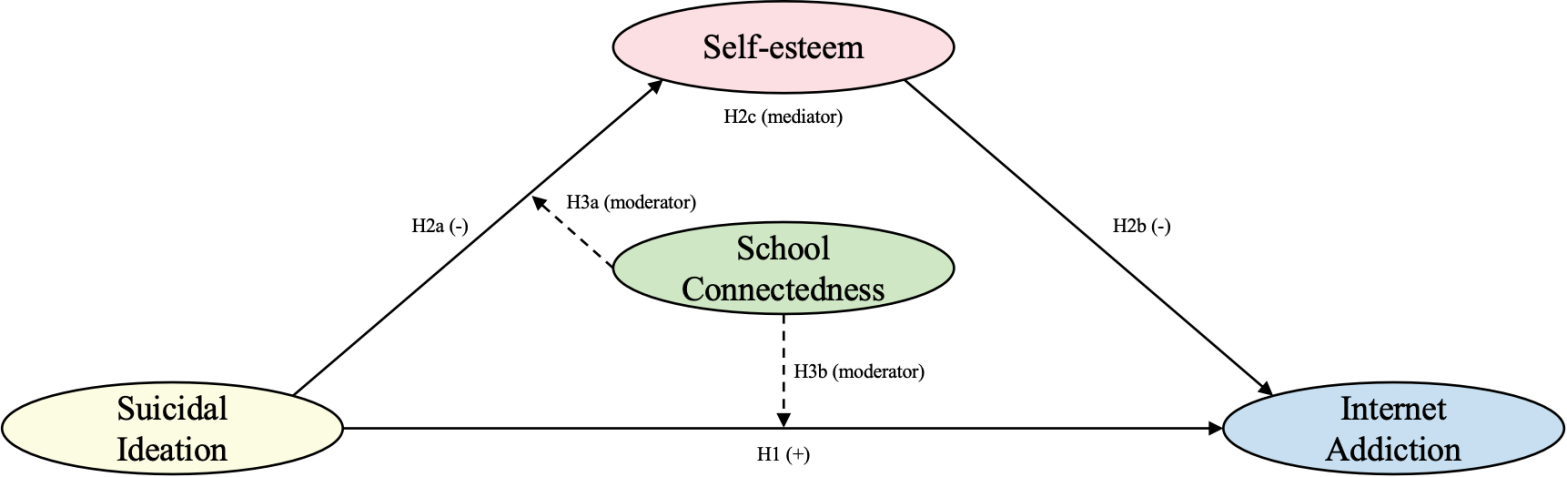
Hypothetical model of the associations between SI and IA among adolescents with SI. H represents hypothesis. The values in parentheses indicate the type of relationship (positive or negative) or the role in the model (moderator or mediator).

Building on the aforementioned research, we propose a hypothetical model based on the CBM-PIU framework (**Figure 1**). We conducted a cross-sectional survey among adolescents with SI in China and used Structural Equation Modeling (SEM) to validate the proposed model. The primary aim of this study is to evaluate the associative pathway from SI to IA. Moreover, we investigate the mediating role of self-esteem (representing proximal internal cognitions) and the moderating role of school connectedness (representing offline social support) in the association between SI and IA.

## 2 Materials and methods

### 2.1 Participants

This study recruited adolescents with SI who visited psychiatric outpatient services at a general hospital and a specialized mental health center in Zhejiang Province, China, between June 2024 and September 2025. Participants were selected from a psychiatric clinic in Hangzhou and a mental health center in Huzhou. Inclusion criteria were as follows (1): adolescents aged 10-19 years (2); diagnosed with suicidal ideation through the Chinese version of the Beck Scale of Suicide Ideation (BSI-CV) (3); willingness to participate in the study and provision of informed consent. Exclusion criteria included (1): adolescents with intellectual disabilities, cognitive impairments, or those unable to comprehend the questionnaire (2); a history of severe neurological conditions (e.g., traumatic brain injury or epilepsy).

Physicians in the psychiatric department identified potential participants and referred them to the research team. Upon receiving the referrals, the research team contacted the adolescents and their guardians to explain the study’s purpose, procedures, and potential risks, and to invite the adolescents to participate. After providing detailed information, written informed consent was obtained from both adolescents and their guardians. All participants completed the questionnaires anonymously in the hospital under the guidance of the research team.

Sample size estimation was conducted according to the minimum sample size guidelines for SEM proposed by Fritz and Mackinnon (45). Previous studies among Chinese adolescents have shown that the path coefficient between SI and self-esteem is approximately 0.19 (32), and that between self-esteem and IA is approximately 0.16 (29). We conservatively assumed that both path coefficients in our model are small. Using bias-corrected bootstrap procedures to validate the mediation effect, the minimum required sample size was calculated to be 462. In this study, 509 adolescents were initially screened, and 462 participants who met the eligibility criteria were ultimately included. This study was approved by the Ethics Committee of the Second Affiliated Hospital of Zhejiang University School of Medicine (Approval No. 2024-0598). All participants and their guardians signed the informed consent form prior to participation.

### 2.2 Measurements

#### 2.2.1 Suicidal ideation

The BSI-CV was used to screen for SI and assess its severity (46). The scale consists of 19 items, each with three response options rated on a three-point scale from 0 to 2. Participants scoring above 0 on either Item 4 (accessing active SI) or Item 5 (accessing passive SI) were classified as having SI. To assess SI severity, the total score is computed by summing the scores of all 19 items, with a range of 0 to 38, where higher scores indicate greater severity. The BSI-CV has demonstrated reliability, validity, and test-retest consistency in assessing SI among Chinese adolescents (46). In this study, the Cronbach’s alpha for the BSI-CV was 0.949.

#### 2.2.2 School connectedness

The School Connectedness Scale (SCS), developed by Yu et al. (47), was used to assess school connectedness. It consists of 10 items across three dimensions: peer support, teacher support, and sense of school belonging. Responses are measured on a five-point Likert scale (1 = completely disagree, 5 = completely agree). Item 2 (“I do not like the teachers at my school”) and 10 (“I have difficulty getting along with other students”) are reverse-coded. After reverse coding, higher total scores indicate greater school connectedness. The Cronbach’s alpha for the SCS was 0.875 among Chinese adolescents, indicating good reliability (48). In this study, the Cronbach’s alpha for the SCS was 0.892.

#### 2.2.3 Self-esteem

The Rosenberg Self-Esteem Scale (RSES), developed by Rosenberg (49), was used in this study to assess self-esteem. The scale consists of 10 items, each rated on a 4-point Likert scale, ranging from 1 (strongly disagree) to 4 (strongly agree). In the Chinese version of the RSES, items 3, 5, 9, and 10 are reverse-scored (50). The total score ranges from 10 to 40, with higher scores indicating more positive self-evaluation and higher levels of self-esteem. The RSES has been widely used in China and demonstrated good reliability, with Cronbach’s alpha ranging from 0.80 to 0.88 among Chinese adolescents (32). In this study, the Cronbach’s alpha for the RSES was 0.932.

#### 2.2.4 Internet addiction

The Internet Addiction Diagnostic Questionnaire (IADQ) (51) was used to assess the degree of IA. The IADQ consists of eight binary items, with participants responding “no” or “yes” to each symptom, which includes preoccupation, tolerance, unsuccessful efforts to limit or stop usage, withdrawal, loss of control over time spent online, risk of losing relationships or opportunities, lying to conceal the extent of involvement, and dysfunctional coping. The total score, ranging from 0 to 8, is calculated by summing the responses. Previous studies have reported Cronbach’s alpha values for the IADQ ranging from 0.62 to 0.66 (2, 52, 53), indicating acceptable reliability. In this study, the Cronbach’s alpha for the IADQ was 0.919.

### 2.3 Statistical analysis

All statistical analyses were performed using IBM SPSS Statistics 27.0 and Amos 24.0. First, descriptive statistics were used for sociodemographic characteristics and study variables. Continuous variables were presented as mean ± standard deviation (SD) or median with interquartile range (IQR), depending on the distribution, while categorical variables were expressed as frequencies and percentages (%). Pearson correlation analysis was used to examine bivariate relationships among variables. Given the cross-sectional, self-reported nature of the data, Common Method Variance (CMV) was rigorously assessed as recommended by Podsakoff et al. (54). Beyond taking specific measures during data collection to minimize CMV (e.g., participant anonymity, reverse-coded items), two statistical approaches were employed: Harman’s single-factor test and a Confirmatory factor analysis (CFA) comparison. For Harman’s test, a variance explanation of less than 40% for the first factor is considered acceptable (55). For the CFA comparison, the fit of the hypothesized measurement model was compared with a single-factor model in which all items loaded onto a single construct (54).

Second, structural equation modeling (SEM) was conducted to examine the hypothesized model (**Figure 1**). Following the “two-step approach” recommended by Anderson and Gerbing (56), we sequentially evaluated the measurement and structural model. CFA was first conducted separately for each of the four latent variables (SI, IA, self-esteem, and school connectedness) to ensure the reliability and validity of the measurement instruments. Following this, SEM was used to examine the relationships among the constructs, with SI as the exogenous variable, IA as the endogenous variable, and self-esteem as a mediator. The model fit was evaluated using five indicators: χ^2^/DF (chi-square to degrees of freedom ratio), Goodness-of-Fit Index (GFI), Incremental Fit Index (IFI), Tucker-Lewis (TLI), and Root Mean Square Error of Approximation (RESEA) with 90% confidence intervals. A good-fitting model is indicated by the normed chi-square between 1 and 3, GFI, IFI, and TLI values greater than 0.90, and an RMSEA with a 90% CI below 0.08 (57). The mediating effect of self-esteem was tested using bootstrapping procedures with 2000 bootstrap samples, and the bias-corrected 95% confidence intervals (CIs) were calculated to assess the significance of the direct and indirect effects. Furthermore, given the IADQ’s binary nature, we conducted a sensitivity analysis by re-evaluating the mediation model using the conventional IADQ sum score.

Finally, for the moderated mediation analysis, the moderating role of school connectedness in the relationship between SI and both self-esteem and IA was examined using PROCESS Model 8 for SPSS (58). In this analysis, age, sex, and family structure were entered as covariates to control for potential confounding. For both the mediation and moderation analyses, the magnitude of the standardized path coefficients was interpreted using Cohen’s guidelines (small ≥ 0.10, medium ≥ 0.30, large ≥ 0.50) (59).

## 3 Results

### 3.1 Demographic and clinical data

A total of 462 adolescents with SI were included in the study. The mean age of participants was 15.61 ± 1.84 years, and 79% of the adolescents were female. The majority of participants (89.6%) were in junior or senior high school. Detailed demographic and psychosocial characteristics are presented in **Table 1**.

**Table 1:** Demographic and psychosocial characteristics of the participants (N = 462).

Characteristics	Mean (SD) or median (IQR) or N (%)
Demographics	
Age (years)	15.61 ± 1.84
Female (%)	365 (79.0%)
Education (%)	
Primary School	17 (3.7%)
Junior High School	190 (41.1%)
Senior High School	224 (48.5%)
College and above	31 (6.7%)
Family structure (%) ^*^	
Extended Family	108 (23.4%)
Nuclear Family	286 (61.9%)
Single-Parent Family	47 (10.2%)
Left-Behind Family	16 (3.5%)
Foster or Adoptive Family	5 (1.1%)
Psychosocial measures	
Suicidal Ideation	19.50 (11.00, 28.00)
Self-esteem	21.00 (16.00, 26.00)
School Connectedness	24.50 (18.00, 32.00)
Internet Addiction	4.00 (1.00, 7.00)

Family structure was categorized based on the living arrangements of children and their caregivers. “Extended Family” refers to households where three generations (grandparents, parents, and children) live together. “Nuclear Family” refers to a family structure in which parents and their children live together. “Single-Parent Family” refers to a family where one parent is responsible for raising the children, with the other parent absent or not involved. “Left-Behind Family” refers to children who live with their grandparents while their parents are working elsewhere. “Fostor or Adoptive Family” includes families where children are raised by foster or adoptive parents, including stepfamilies or guardianship situations.

### 3.2 Preliminary analyses and common method variance

Pearson correlation analysis revealed significant correlations between SI, self-esteem, school connectedness, and IA. The detailed correlation matrix is presented in **Table 2**.

**Table 2:** Correlation matrix of key variables.

Variables	1	2	3	4
1. Suicidal ideation	1			
2. Self-esteem	-0.441^***^	1		
3. School connectedness	-0.119^***^	0.217^***^	1	
4. Internet addiction	0.429^***^	-0.534^***^	-0.234^***^	1

The values represent Pearson correlation coefficients between the key variables. Negative values indicate negative associations, while positive values indicate positive associations between the variables. The asterisks of the coefficients in the upper right corner denote statistical significance. *** indicating p<0.001.

Regarding CMV, the exploratory factor analysis conducted for Harman’s single-factor test yielded four distinct factors, each with an eigenvalue greater than 1. The first factor accounted for only 30.86% of the total variance, falling well below the 40% threshold. Furthermore, the CFA model comparison demonstrated that the hypothesized four-factor model fit the data well (χ^2^ = 988.69, GFI = 0.905, IFI = 0.976, TLI = 0.975, RMSEA = 0.027), whereas the single-factor model exhibited a poor fit (χ^2^ = 5059.85, GFI = 0.421, IFI = 0.600, TLI = 0.577, RMSEA = 0.113). The substantial deterioration in fit indices (Δχ^2^ = 4071.16, ΔGFI = 0.484, ΔIFI = 0.376, ΔTLI = 0.398, ΔRMSEA = -0.086) confirms that a single common factor cannot account for the data’s variance. Collectively, these results indicate that CMV was not a threat in this study.

### 3.3 Examination of the measurement model

All measurement models demonstrated good fit with the data (**Supplementary Table 1**). Reliability was evaluated using factor loadings and construct reliability (CR) values. According to Fornell and Larcker (60), factor loadings greater than 0.60 and CR values greater than 0.70 indicate good reliability. In this study, the factor loadings ranged from 0.617 to 0.855, and CR values for all constructs ranged from 0.819 to 0.951, indicating good reliability for the latent constructs.

Convergent validity was assessed by examining the average variance extracted (AVE) for each construct. All AVE values exceeded 0.50, ranging from 0.505 to 0.674, indicating satisfactory convergent validity (60). Discriminant validity was assessed by comparing the AVE for each latent variable with the square of the Pearson correlation coefficient between each pair of latent variables. The AVE for each latent variable was larger than the squared correlation with any other latent variable, indicating satisfactory discriminant validity (60). Detailed results of the CFA are presented in **Supplementary Table 1**, while the results of the discriminant validity analysis are shown in **Table 3**.

**Table 3:** Average variance extracted (AVE) and discriminant validity analysis.

Variables	1	2	3	4
1. Suicidal ideation	**0.505**			
2. Self-esteem	0.194	**0.586**		
3. School connectedness	0.014	0.047	**0.674**	
4. Internet addiction	0.184	0.285	0.055	**0.591**

Diagonal values (bolded) represent the Average Variance Extracted (AVE) values of each latent variable. The off-diagonal values represent the squared Pearson correlation coefficients between each pair of latent variables.

### 3.4 Examination of the structural model

**Figure 2:**
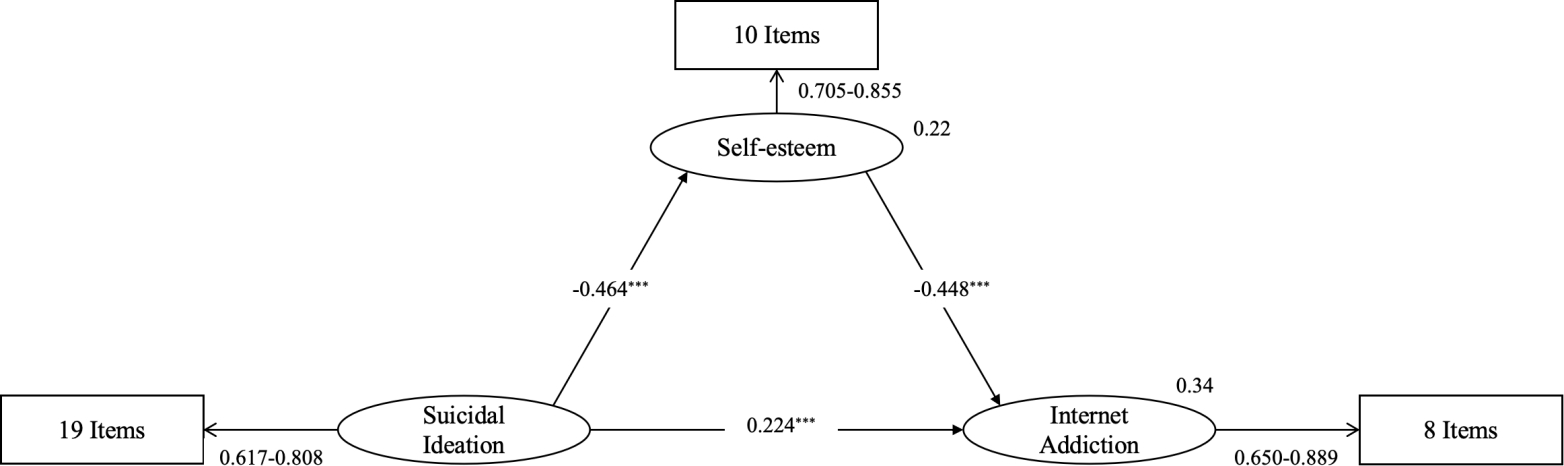
Mediation model of the association between suicidal ideation and internet addiction with self-esteem as the mediator. The ellipses represent latent variables, while the rectangles represent observed variables. The numbers along the paths indicate standardized path coefficients. The measurement models show the range of factor loading. The number in the upper-right corner of the latent variable represents R2 (coefficient of determination). ***indicates a p-value <0.001.

The structural model demonstrated a good fit to the observed data, with the following goodness-of-fit indices: χ^2^/df = 1.447, GFI = 0.905, IFI = 0.973, TLI = 0.972, and RESEA = 0.031 with a 90% confidence interval of 0.027 to 0.036. SI was significantly positively associated with IA (standardized coefficient = 0.224, p < 0.001, indicating a small-to-medium effect size), confirming Hypothesis 1. SI was also negatively associated with self-esteem (β = -0.464, *p* < 0.001, representing a medium-to-large effect), confirming Hypothesis 2a, and self-esteem was further negatively associated with IA (β = -0.448, p < 0.001, also representing a medium-to-large effect), confirming Hypothesis 2b. Detailed results are shown in **Figure 2**.

### 3.5 Analysis of the mediation effects of self-esteem

The bootstrapped standardized total effect of SI on IA was 0.432, with a 95% bias-corrected confidence interval (CI) ranging from 0.349 and 0.509. The direct effect from SI to IA was 0.224, with a 95% CI ranging from 0.127 to 0.316. The indirect effect was 0.208 (95% CI: 0.154-0.271). Since neither the direct nor the indirect effect CI includes zero, this suggests that self-esteem partially mediates the relationship between SI and IA, confirming Hypothesis 2c (61). The indirect effect accounts for 48.14% of the total effect. The detailed results of the mediation effects are presented in **Table 4**. Furthermore, in the sensitivity analysis utilizing the conventional IADQ sum score, the indirect effect of self-esteem remained highly significant (indirect effect = 0.205, 95% CI: 0.154, 0.264), confirming the robustness of these findings (**Supplementary Table 2**).

**Table 4:** Pathway estimates and bootstrapped confidence intervals for the association between suicidal ideation and internet addiction.

Relationship	Effect type	Point estimates	Bootstrapping bias-corrected 95% CI		Ratio (%)
			Lower bounds	Upper bounds	
SI→SE→IA	Direct Effect	0.224	0.127	0.316	51.85
	Indirect Effect	0.208	0.154	0.271	48.15
	Total Effect	0.432	0.349	0.509	/

The table presents the bootstrapped point estimates and 95% bias-corrected confidence intervals (CI) for self-esteem (SE) as a mediator in the association between suicidal ideation (SI) and internet addiction (IA). The direct, indirect, and total effects are reported, along with the corresponding ratio of the indirect and direct effects to the total effect.

Given the skewed gender distribution (79.0% female), an exploratory multi-group SEM analysis was conducted as a robustness check. Critical ratios for parameter differences revealed no significant gender variations across the core structural associations: between SI and self-esteem (z = 0.316, p > 0.05), self-esteem and IA (z = -1.890, p > 0.05), and SI and IA (z = -1.444, p > 0.05). These results confirm the structural invariance across gender.

### 3.6 Verification of the moderating effects of school connectedness

**Figure 3:**
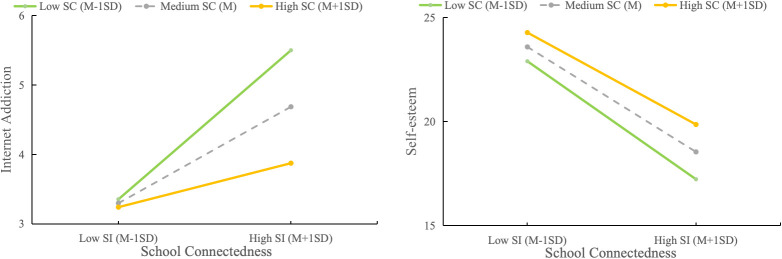
Interaction between suicidal ideation (SI) and school connectedness (SC) in relation to self-esteem (SE) (Left) and internet addiction (IA) (Right). The slopes represent the strength and direction of the relationship between SI and the outcomes (SE and IA) at each level of SC. The interaction between SI and SE is not significant (p=0.202), while the interaction between SI and IA is significant (p=0.001). The analysis was adjusted for age, sex, and family structure.

The moderation analysis revealed a significant interaction between school connectedness and SI on IA (β = -0.005, SE = 0.002, t = -3.258, p = 0.001, 95% CI: -0.008 -0.002), indicating a small but statistically significant effect and confirming Hypothesis 3b. However, the interaction between school connectedness and SI was not significantly associated with self-esteem (β = 0.004, SE = 0.003, t = 1.273, p = 0.202, 95% CI = -0.002 – 0.010), indicating that Hypothesis 3a was not supported (**Table 5**). Further simple slope analyses showed that school connectedness moderated the relationship between SI and IA, with significant associations at lower levels of school connectedness (β = 0.120, SE = 0.020, p < 0.001, 95% CI = 0.081 – 0.159, small effect) and medium levels of school connectedness (β = 0.078, SE = 0.015, p < 0.001, 95% CI = 0.048 – 0.107, small effect). However, the effect at higher levels of school connectedness was not significant (β =0.035, SE = 0.020, p = 0.073, 95% CI = -0.003 – 0.074) (**Figure 3**).

**Table 5:** Regression analysis of the moderation model for self-esteem and internet addiction.

Variable	M: Self-esteem			Y: Internet addiction		
	β	SE	t	β	SE	t
Constant	23.985	2.587	9.271^***^	11.176	1.332	8.392^***^
SI	-0.282	0.029	-9.856^***^	0.078	0.015	5.217^***^
SE	/	/	/	-0.209	0.022	-9.462^***^
SC	0.117	0.029	3.975^***^	-0.051	0.014	-3.590^***^
SI × SC	0.004	0.003	1.277	-0.005	0.002	-3.258^***^
R^2^	0.003			0.015		
F	1.631			10.614		
P	0.202			0.001		

The table shows the unstandardized coefficient (β), standard errors (SE), t-values, and p-values. The model includes suicidal ideation (SI) as the predictor, self-esteem (SE) as the mediator, and school connectedness (SC) as the moderator. The model was adjusted for age, sex, and family structure. *p<0.05, ***p<0.001.

## 4 Discussion

This study focuses on IA among adolescents with SI, exploring the mechanism underlying the association between SI and IA, guided by the CBM-PIU framework. Using SEM, we examine the mediating role of self-esteem (representing proximal maladaptive cognitions) in the association between SI and IA, and further investigate how school connectedness (representing offline social support) moderates the association of SI with both self-esteem and IA. To the best of our knowledge, this is the first study to specifically explore the associative pathway from SI to IA in adolescents with SI, providing novel insights into the mechanism of IA in this vulnerable group.

This study found that SI is positively associated with IA, consistent with previous research (62, 63). This finding strongly supports the CBM-PIU, which posits that underlying psychopathology acts as a critical distal vulnerability associated with a higher likelihood of IA (18). Immersion in the internet is not only viewed as an addiction but also as a coping strategy to escape negative emotions and thoughts (17, 21). Adolescents with SI often experience psychological distress, such as feelings of burden and hopelessness, due to the gap between their current situations and their expectations (64, 65). According to the Dual-System Model of Suicidality, these expectational gaps motivate individuals to seek relief (66). The internet, with its sense of control, validation, and achievement, may offer temporary respite from these feelings, which is associated with a greater tendency for internet immersion among adolescents with SI (17).

In addition to the direct association between SI and IA, SI is also indirectly linked to higher levels of IA through its negative association with self-esteem, which serves as a significant mediator in this relationship. These findings partially support previous research on the bidirectional relationship between self-esteem and SI (32), demonstrating that self-esteem is not only negatively associated with SI, but SI is also significantly negatively associated with self-esteem. Adolescents with high levels of SI tend to exhibit negative biases and maladaptive internal schemas, which are often accompanied by feelings of worthlessness and self-criticism, and are reflected in low self-esteem (34). In turn, as posited by the CBM-PIU, adolescents with these maladaptive self-cognitions may struggle to obtain real-world validation. This deficit is associated with a tendency to seek online compensation and, subsequently, more severe IA (18). Therefore, cognitive restructuring targeting self-esteem can be a key intervention target for reducing IA in adolescents with SI and may also provide additional benefits by alleviating their SI, given the bidirectional relationship between self-esteem and SI.

In this study, school connectedness was found to moderate the association between SI and IA, with higher levels of school connectedness associated with a weaker relationship. This finding provides empirical support to the CBM-PIU and aligns with previous research suggesting that greater school-based support, such as peer support, is inversely associated with adolescent IA (67). According to the interpersonal theory of suicide, thwarted belongingness, characterized by loneliness, fewer social connections, and social withdrawal, is a key risk factor for passive suicidal ideation (68, 69). Adolescents with SI may obtain a sense of belonging and emotional validation from school-based support, providing an alternative avenue for interpersonal connection. Additionally, peer support is closely linked to effective coping strategies (70). Adolescents with SI who experience high levels of peer support are more likely to adopt adaptive problem-solving coping strategies for real-life conflicts. This behavior, in turn, is associated with a reduced tendency to use the internet for escapism.

It is noteworthy that although this study found school connectedness significantly moderates the association between SI and IA, the magnitude of this moderating coefficient was relatively small (β = -0.005). Importantly, our simple slopes analysis demonstrates that the protective buffering effect against the SI-IA link requires relatively high levels of school connectedness (e.g., +1SD) to render the association non-significant. This may be because, although school-based social support is valuable, internet-based support offers adolescents a sense of security and anonymity, allowing them to form relationships without fear of immediate judgment or rejection (71). The internet, therefore, can be particularly appealing to adolescents with SI, especially when faced with negative life events, and may function as a safer alternative. These findings suggest that while school connectedness is a potential intervention target, enhancing school connectedness should not be viewed as a standalone clinical strategy. Additional strategies are needed to enhance adolescents’ positive experiences within school support systems and to encourage greater engagement with them. For example, nudge strategies, such as providing social reference points and giving individuals feedback on behavior, have demonstrated promising benefits in guiding individuals toward healthier decisions, particularly in areas like eating habits and physical activity, and can be easily scaled for broader implementation (72, 73). Future research could integrate nudge strategies into school-connectedness-targeted interventions to more effectively address IA among adolescents with SI.

Although school connectedness moderated the association between SI and IA, it did not moderate the association between SI and self-esteem. We hypothesize that this discrepancy may be explained by an “ecological level mismatch”. Social ecological models of health promotion posit that health outcomes are shaped by interactions across multiple nested levels of influence, including individual, interpersonal, and organizational environments (74). A lack of alignment between a targeted outcome and a protective factor can substantially limit the protective efficacy of the factor (75). Specifically, the link between SI and low self-esteem is characterized primarily by maladaptive cognitions (e.g., individual cognitive biases) operating at the individual level (34). School connectedness, conversely, provides support at the interpersonal level. As an external interpersonal resource, school connectedness may be insufficient on its own to address internal cognitive distortions. In contrast, IA among adolescents with SI is primarily associated with interpersonal-level deficits, such as unmet social needs and a lack of external validation (17). Because school connectedness directly provides offline social resources to address these deficits, it aligns with the ecological level of IA and thereby functions as a moderator. Collectively, these findings indicate that protective factors may demonstrate stronger protective associations when aligned with the specific ecological level of the targeted outcome.

This study offers several clinical implications. First, the significant positive association between SI and IA highlights adolescents with SI as a highly vulnerable population for co-occurring IA, emphasizing the need for routine clinical screening in this group. Second, the robust indirect association through self-esteem suggests that maladaptive cognitions serve as a crucial theoretical link between SI and IA. Because IA may act as a coping strategy for underlying psychosocial distress among adolescents with SI (17, 21), purely behavioral restrictions might remove a perceived coping tool and inadvertently leave them at risk for alternative, more harmful escape behaviors, including suicide attempts (76). Consequently, rather than solely restricting behavior, comprehensive interventions should address the underlying psychological drivers. Integrating individual-level interventions, such as cognitive behavioral therapy aimed at restructuring self-esteem, can help resolve these root maladaptive cognitions and reduce the need for online coping. Finally, school connectedness demonstrated only a small moderating role in the association between SI and IA and failed to moderate the link between SI and self-esteem. This suggests that interventions relying solely on school connectedness may yield limited clinical benefits, likely due to an “ecological level mismatch” and the unique sense of security and anonymity the internet provides over offline interactions. Therefore, efforts targeting school connectedness should incorporate proactive strategies to enhance adolescents’ positive experiences and active engagement with school support systems. Crucially, to optimize intervention efficacy, the selected therapeutic strategies should closely align with the specific ecological level of the pathways driving IA among adolescents with SI.

Our study has several limitations that should be acknowledged. First, regarding the study design, the cross-sectional, self-reported nature of the data does not allow us to establish causal or bidirectional relationships, nor can it capture dynamic changes in the variables over time. Although we statistically addressed CMV, self-report bias remains. Future research should employ longitudinal or multi-wave designs to confirm the directionality of these associations, alongside multi-informant approaches (e.g., clinician or parental reports) to validate the findings. Second, regarding measurement, while the binary IADQ minimized respondent burden for this high-risk clinical sample, it limits sensitivity in capturing addiction severity gradients compared to Likert-type scales and potentially nuanced associations. Moreover, the unusually high internal consistency (Cronbach’s alpha = 0.919) observed in this study likely reflects the clustering of severe symptoms characteristic of this specific clinical population. It raises potential psychometric concerns, such as extreme response bias. Although our CFA supported the scale’s unidimensionality, future studies should employ continuous measures to capture nuanced symptom profiles. Third, regarding generalizability, our sample characteristics restrict broader applicability. Convenience sampling from only two psychiatric outpatient clinics in a single Chinese province excludes non-clinical adolescents with SI in the community, such as those who avoid seeking help due to stigmatization (77). Furthermore, although our multi-group analysis indicated structural invariance across genders, the unbalanced gender distribution (79.0% female) may still obscure gender heterogeneity, as previous studies suggest these associations might be stronger in males (78). The potential influence of broader cultural contexts on external support-seeking was also unexplored. Future studies require more diverse, community-based samples with balanced gender ratios across different regions. Finally, regarding unmeasured variables, other psychosocial factors may also be essential for understanding the complex relationship underlying the association between SI and IA, which warrants deeper exploration in future studies. Despite these limitations, our study provides valuable preliminary insights into the associative relationships among these factors in adolescents with SI, offering a theoretical foundation for future longitudinal research and intervention studies.

## 5 Conclusion

In this study, SI was significantly positively associated with IA among adolescents with SI. Self-esteem partially mediated the relationship between SI and IA. School connectedness significantly moderated the association between SI and IA, with higher connectedness associated with a weaker link. However, school connectedness did not moderate the association between SI and self-esteem. These findings explore the associative patterns underlying IA in adolescents with SI and identify potential intervention targets. Ultimately, this study provides preliminary theoretical insights that can inform future longitudinal studies and targeted clinical interventions.

## Data Availability

The raw data supporting the conclusions of this article will be made available by the authors, without undue reservation.
