# Supplementary material for: Internet addiction in adolescents with suicidal ideation: the role of self-esteem and school connectedness
Source: Front Psychiatry. 2026 Apr 1;17:1775949. doi: 10.3389/fpsyt.2026.1775949 (PMC13080606; doi:10.3389/fpsyt.2026.1775949)
Supplement: Supplementary file 1 [file DataSheet1.pdf]

Supplementary Material

1 Supplementary Tables

Supplementary Table 1. Confirmatory Factor Analysis (CFA) Results: Model Parameter Estimates and Fit Indices

Construct	Indicator	Model Parameter Estimates				Covergent Validity				Model Fit Indices						
		Undstandardized FL	S.E.	C.R.	P	Standardized FL	SMC	C.R.	AVE	χ^2	DF	χ^2/DF	GFI	IFI	TLI	RMSEA (90% CI)
Suicidal Ideation	SI1	1	/	/	/	0.756	0.571	0.951	0.505	305.83	152	2.012	0.932	0.969	0.965	0.047 (0.039, 0.054)
	SI2	0.856	0.054	15.846	***	0.709	0.487									
	SI3	1.08	0.067	16.024	***	0.716	0.486									
	SI4	0.866	0.058	15.006	***	0.676	0.456									
	SI5	0.778	0.052	15.106	***	0.680	0.653									
	SI6	1.163	0.071	16.389	***	0.731	0.455									
	SI7	1.173	0.072	16.197	***	0.723	0.506									
	SI8	0.737	0.05	14.745	***	0.665	0.649									
	SI9	0.991	0.061	16.36	***	0.730	0.448									
	SI10	0.989	0.064	15.56	***	0.698	0.381									

	SI11	0.927	0.06	15.545	***	0.697	0.537									
	SI12	0.77	0.051	14.981	***	0.675	0.503									
	SI13	1.457	0.079	18.422	***	0.808	0.513									
	SI14	0.865	0.058	14.98	***	0.675	0.457									
	SI15	1.071	0.067	15.891	***	0.711	0.462									
	SI16	0.918	0.05	18.353	***	0.805	0.534									
	SI17	0.981	0.066	14.833	***	0.669	0.523									
	SI18	0.687	0.051	13.567	***	0.617	0.443									
	SI19	1.188	0.072	16.438	***	0.733	0.532									
Self-Esteem	SE1	1	/	/	/	0.843	0.711	0.934	0.586	66.338	35	1.895	0.973	0.989	0.986	0.044 (0.027, 0.060)
	SE2	0.972	0.048	20.352	***	0.787	0.528									
	SE3	0.915	0.049	18.688	***	0.743	0.619									
	SE4	0.94	0.04	23.268	***	0.855	0.552									
	SE5	0.837	0.046	18.319	***	0.733	0.731									
	SE6	0.879	0.04	21.735	***	0.82	0.537									
	SE7	0.833	0.046	18.039	***	0.725	0.673									
	SE8	0.81	0.047	17.171	***	0.7	0.526									

	SE9	0.876	0.051	17.324	***	0.705	0.49									
	SE10	0.904	0.05	18.11	***	0.727	0.496									
	SC2	1	/	/	/	0.808	0.652									
School Connectedness (Teacher Support)	SC5	0.816	0.046	17.814	***	0.798	0.637									
	SC8	0.901	0.048	18.714	***	0.847	0.717									
	SC1	1	/	/	/	0.865	0.748									
School Connectedness (Peer Support)	SC4	0.946	0.046	20.763	***	0.797	0.636	0.954	0.674	68.301	32	2.134	0.972	0.987	0.981	0.050 (0.033, 0.066)
	SC7	1.067	0.052	20.426	***	0.789	0.622									
	SC10	1.308	0.054	24.173	***	0.88	0.775									
	SC3	1	/	/	/	0.82	0.672									
School Connectedness (School Belongingness)	SC6	1.038	0.06	17.224	***	0.774	0.599									
	SC9	1.138	0.062	18.325	***	0.828	0.686									
	IA1	1	/	/	/	0.814	0.662									
	IA2	0.985	0.05	19.809	***	0.802	0.644									
Internet Addiction	IA3	0.934	0.051	18.473	***	0.763	0.582	0.919	0.591	66.998	20	3.35	0.965	0.979	0.971	0.071 (0.053, 0.091)
	IA4	0.936	0.051	18.52	***	0.764	0.584									
	IA5	0.735	0.049	15.146	***	0.655	0.429									

IA6	0.733	0.049	15.014	***	0.65	0.423
IA7	1.091	0.048	22.96	***	0.889	0.79
IA8	0.929	0.049	19.088	***	0.781	0.61

Note: Unstandardized factor loading (FL), standard errors (S.E.), critical ratio (C.R.), and P-values are provided. Composite Reliability and Average Variance Extracted (AVE) are calculated based on standardized FLs. Model fit indices include χ^2/DF (chi-square to degrees of freedom ratio), Goodness-of-Fit Index (GFI), Incremental Fit Index (IFI), Tucker-Lewis (TLI), and Root Mean Square Error of Approximation (RMSEA) with 90% confidence intervals.

*** indicates a p-value <0.001.

Supplementary Table 2. Pathway Estimates and Bootstrapped Confidence Intervals for the Relationship between Suicidal Ideation and Internet Addiction (Sum Score Model)

Relationship	Effect Type	Point Estimates	Bootstrapping Bias-corrected 95% CI		Ratio (%)
			Lower Bounds	Upper Bounds	
	Direct Effect	0.231	0.138	0.318	52.98
SI→SE→IA	Indirect Effect	0.205	0.154	0.264	47.01
	Total Effect	0.436	0.357	0.507	/

Note: The table presents the bootstrapped point estimates and 95% bias-corrected confidence intervals (CI) for self-esteem (SE) as a mediator in the relationship between suicidal ideation (SI) and internet addiction (IA). The direct, indirect, and total effects are reported, along with the corresponding ratio of the indirect and direct effects to the total effect.
